# Demographics of Astrocytoma in Central Texas: The Interaction Between Race, Histology, and Primary Tumor Site

**DOI:** 10.7759/cureus.9676

**Published:** 2020-08-11

**Authors:** Damir Nizamutdinov, Samantha Dayawansa, Ekokobe Fonkem, Jason H Huang

**Affiliations:** 1 Neurosurgery, Baylor Scott & White Medical Centre, Temple, USA; 2 Neurosurgery, Baylor Scott & White Medical Center, Temple, USA; 3 Neurology, St. Joseph’s Medical Center, Phoenix, USA

**Keywords:** epidemiology, astrocytoma, demographics, prognostic factors

## Abstract

Introduction

The epidemiological factors surrounding astrocytoma and gliomas have been studied with little avail. Even limited conclusions have not been reached in spite of significant past research efforts. Ionizing radiation is currently one of the only factors consistently associated with glioma formation. Studies in an attempt to link environmental and occupational exposures with brain neoplasms have continued to produce inconsistent results. This study aims to explore the distribution and epidemiology of astrocytomas within a Central Texas patient population in order to elucidate any possible differences in epidemiologic and prognostic factors based on race, histology, and primary tumor site.

Methods

Eight hundred forty-five clinical cases with the diagnosis of astrocytoma were retrospectively obtained from the tumor registry of the Scott & White Integrated Healthcare System from 1976 to 2014. We investigated the effects of gender, race, tumor histology, tumor site, treatment methods, and mortality of this cohort of patients in Central Texas.

Results

Prevalence data echoes that of the national epidemiology in that among our sample, White individuals had the highest prevalence (n=666, 78.8%), followed by Hispanics (n=94, 11.1%) and Black individuals (n=78, 9.2%). White patients had higher rates of parietal lobe (6.6% vs. 0.6%, p<0.01), brain overlapping (6.8% vs. 0.0%, p<0.01), and brainstem (5.9% vs. 1.7%, p=0.02) tumors. Black patients had higher rates of tumors located in brain (not otherwise specified) (35.9% vs. 15.7%, p<0.01) and cerebellum (33.3% vs. 5.6%, p<0.01). Hispanic patients had higher rates of tumor located in the temporal lobe (31.9% vs. 22.8%, p<0.05) and brain (not otherwise specified) (28.7% vs. 16.1%, p<0.01). Hispanics had the largest proportion of deaths (72.3% vs. 38.0%, p<0.01) when compared to the remainder of the sample, followed by White individuals (39.6% vs. 49.7%, p=0.02) and Black individuals (21.8% vs. 43.8%, p<0.01).

Conclusions

Discrepancies in mortality rates amongst various racial groups may be due to a number of factors. Primary tumor site and histology seem to indeed play a role in mortality and may present variably between ethnic groups. Mortality is also influenced by race, genetic predisposition, environmental and occupational exposure, and access to healthcare.

## Introduction

Astrocytomas are defined as clusters of aberrant astrocyte growth within the central nervous system (CNS). They are a subset of glial tumors, which also include gliomas, glioblastomas, and gangliogliomas. While glioblastoma accounts for the majority of gliomas, astrocytomas and glioblastoma combined account for about 75% of all gliomas. Gliomas account for 28% of all brain tumors and 80% of malignancies. Most of these tumors (60.9%) tend to occur in the frontal, temporal, parietal, and occipital lobes combined and only a marginal proportion are found outside the brain [1]. Astrocytomas are classified as either diffuse or pilocytic. Diffuse astrocytomas can further be delineated based on histopathological analysis as fibrillary, gemistocytic, or protoplasmic. These diffuse variants invade large areas of surrounding neural tissue. The most common of these is the fibrillary subtype, which is identified by the presence of fibrillary astrocytes dispersed within a loose, microcystic tumor matrix. Gemistocytic astrocytomas are comprised of gemistocytes that can be identified by their eosinophilic cytoplasm, round cell processes, and cytoplasmic accumulation of glial fibrillary acidic protein (GFAP) [2,3]. The protoplasmic subtype is the rarest and tends to primarily affect the cortex. Given their rarity, the literature only consists of a limited number of case reports and small series, one of which documents a diffuse and non-localized form in a three-year-old female with no identifiable primary site [3-5]. However, it has been suggested that their prognosis is comparable to that of the fibrillary subtype [3]. Pilocytic astrocytomas are indolent, well-circumscribed tumors that have an associated cystic component and tend to occur almost exclusively and most commonly in children and young adults. They are the most common glioma in children, however, there are instances where they have been seen in older individuals [6,7]. When seen in adult populations, pilocytic astrocytomas are most commonly of anaplastic characterization and exceptionally rare. Given their rarity and paucity of data, adult anaplastic pilocytic astrocytomas are currently not explicitly recognized as a distinct entity, yet available data suggest they behave differently from more common high-grade glioma [8]. Pilocytic variants are most likely to affect the cerebellar hemispheres and areas around the third ventricle but also are encountered in the cerebral hemispheres [9,10]. Histologically they consist of a scant intercellular fibrillary matrix and express GFAP [2,3]. Additionally, pilocytic astrocytomas are associated with Rosenthal fibers, which historically have been used to differentiate them from other astrocytoma types [11]. Recently, however, a relatively new subtype known as pilomyxoid has also been associated with the presence of Rosenthal fibers, thus perhaps diminishing their distinguishing property [11,12]. However, much like pilocytic variants, the majority of pilomyxoid astrocytoma are restricted to the hypothalamus and chiasmatic regions and occur in children under the age of four [13]. They have been known to be slightly more aggressive than their pilocytic counterparts [14].

Struggles continue to exist today in the study of astrocytomas [15]. This is partly due to the lack of consensus definitions regarding glioma subtypes and discrepancies in data collection between various sources resulting in complications when comparing prevalence rates. Many studies have been dedicated to elucidating the epidemiological factors surrounding astrocytoma and gliomas in general with little avail. Few consistent findings have ever been made despite several decades of research. Ionizing radiation is one of the only factors that has consistently been associated with glioma formation. Other environmental exposures, such as cell phone use, have been proved to show no association with disease onset. Studies looking at occupational exposure have also proven to be inconsistent in their findings [16]. However, mutations in several genomic loci have been linked to glioma formation and could provide a link to racial discrepancies in prevalence [17].

This study aims to explore the distribution and epidemiology of astrocytomas within a Central Texas patient population in order to elucidate any possible differences in epidemiologic factors based on primary tumor location, histology, and ethnicity.

## Materials and methods

Sources of data and study population

All human investigations were performed after approval by the Institutional Review Board and in accordance with an assurance filed with and approved by the U.S. Department of Health and Human Services. Eight hundred and forty-five clinical cases of astrocytoma were obtained from the tumor registry of the Scott & White Integrated Healthcare System from 1976 to 2014. Race was categorized as White, Hispanic, Black, and non-Hispanic, with White vs. non-White, Black vs. non-Black, and Hispanic vs. non-Hispanic also examined. Our categorization of tumor grade fell into three groups as determined by histopathological studies: grade I, grade II, and grade III. Prevalence was calculated based on census data for Bastrop, Bell, Blacno, Burnet, Coryell, Gillespie, Hays, Lampasas, Lee, Llano, Falls, McLennan, Milam, Travis, and Williamson counties. Prevalence per 100,000 for patients diagnosed between 1976 and 1985 was calculated using 1980 census data, 1986 and 1995 using 1990 census data, 1996 and 2005 using 2000 census data, and 2006 and 2014 using 2010 census data. The average incidence for all years from 1976 to 2014 was 1.3 per 100,000.

Data analysis

The data were incorporated from an excel file into SAS, v9.2 (SAS Institute, Cary, NC) and R, v2.15.1 (The R Foundation for Statistical Computing, Vienna, Austria) to be analyzed for a number of variables. Descriptive statistics, including frequencies and percentages, were calculated to describe patient characteristics, tumor location, and mortality among ependymoma cases. Comparisons for mortality among locations were examined, overall and pairwise, using two-sample proportion tests. Bivariate analyses assessed underlying differences in characteristics and survival among patients. Chi-square analysis (Fisher’s exact tests for small expected cell counts) was employed to compare categorical variables, and the nonparametric Wilcoxon rank-sum and Kruskal-Wallis tests were employed for continuous measures. A type I error of α=0.05 was assumed throughout.

## Results

Demographics and tumor locations

A total of 845 patients were included in the study analysis. The demographics of the study population and the tumor characteristics have been included in Table 1.

**Table 1:** Patient demographics and tumor characteristics (n=845) Table 1 illustrates the demographics of the patient population included in the study, primary tumor site location, and tumor histology. NOS: not otherwise specified.

Patient Characteristics	Study Sample (n=845)
Mean Age at Diagnosis (SD):	35.4 (22.5)
Median Age (Min-Max):	35.0 (0-87)
Male	432 (51.1%)
Female	413 (48.9%)
Race/Ethnicity	
White	666 (78.8%)
Hispanic	94 (11.1%)
Black	78 (9.2%)
Other/Unknown	7 (0.8%)
Primary Tumor Site	
Temporal lobe	201 (23.8%)
Frontal lobe	178 (21.1%)
Parietal lobe	45 (5.3%)
Occipital lobe	21 (2.5%)
Brain, overlapping	45 (5.3%)
Brain, NOS	148 (17.5%)
Cerebrum	83 (9.8%)
Cerebellum, NOS	69 (8.2%)
Brain stem	42 (5.0%)
Ventricle, NOS	11 (1.3%)
Posterior cranial	1 (0.1%)
Fourth ventricle	1 (0.1%)
Histology	
Astrocytoma, NOS	394 (46.6%)
Astrocytoma, pilocytic	168 (19.9%)
Astrocytoma, anaplastic	154 (18.2%)
Astrocytoma, fibrillary	56 (6.6%)
Astrocytoma, gemistocytic	38 (4.5%)
Xanthoastrocytoma, pleomorphic	24 (2.8%)
Oligoastrocytoma, anaplastic	4 (0.5%)
Astrocytoma, protoplasmic	4 (0.5%)
Astrocytoma, juvenile	2 (0.2%)
Astrocytoma, diffuse	1 (0.1%)

White and Black astrocytoma patients had significantly lower mortality when compared with non-white (39.6% vs. 49.7%, p=0.02), and non-black patients (21.8% vs. 43.8%, p<0.01), respectively. Hispanic patients carried a greater risk of death (72.3% vs. 38.0%, p<0.01) when compared with the non-Hispanic population. 

The overall mean incidence in Central Texas counties based on cases presenting to Scott & White Clinic was calculated to be 1.3 per 100,000. Specifically, the incidence of the White population at 1.61, the Hispanic population at 0.56, the Black population at 1.06, and other race at 0.15. ANOVA test revealed a significant difference in the prevalence between White and Hispanic (t=-2.843, p<0.01), and White and other race (t=-2.459, p=0.01505). The difference between the White and Black populations did not reach statistical significance (t=1.483, p=0.14020).

Race and primary tumor site

White patients had higher rates of astrocytoma located in parietal lobe (6.6% vs. 0.6%, p<0.01), brain overlapping (6.8% vs. 0.0%, p<0.01), and brainstem (5.9% vs. 1.7%, p=0.02), while lower rates of brain, not otherwise specified (NOS) (13.5% vs. 32.4%, p<0.01), and cerebellum (5.7% vs. 17.3%, p<0.01) astrocytoma locations when compared with locations in non-White patients (Table 2).

**Table 2:** Statistics of race, tumor site, and histology Table 2 shows patients stratified by race, primary tumor site, and histology (n=845) and results of statistical analysis. NOS: not otherwise specified.

Variable	Total				White			Hispanic			Black			Other		
Primary Tumor Site	N	% Alive	Chi Square	p	N (%)	Chi Square	p	N (%)	Chi Square	p	N (%)	Chi Square	p	N (%)	Chi Square	p
Temporal lobe	201	60.7	0.6624	0.4157	168 (25.23)	3.5874	0.0582	30 (31.92)	3.8542	0.0496	0 (0)	26.8205	<0.0001	3 (42.86)	1.416	0.2341
Frontal lobe	178	37.64	39.2827	<0.0001	143 (21.47)	0.3123	0.5763	21 (22.34)	0.1035	0.7477	14 (17.95)	0.5019	0.4787	0 (0)	1.8837	0.1699
Parietal lobe	45	28.89	16.8172	<0.0001	44 (6.61)	10.2352	0.0014	1 (1.06)	3.8098	0.051	0 (0)	4.8337	0.0279	0 (0)	0.397	0.5286
Occipital lobe	21	52.38	0.3024	0.5824	20 (3)	3.4783	0.0622	1 (1.06)	0.8817	0.3477	0 (0)	2.19	0.1389	0 (0)	0.1799	0.6715
Brain, overlapping	45	66.67	1.3926	0.238	45 (6.76)	12.7749	0.0004	0 (0)	5.9493	0.0147	0 (0)	4.8337	0.0279	0 (0)	0.397	0.5286
Brain, NOS	148	68.92	8.4362	0.0037	90 (13.51)	34.8413	<0.0001	27 (28.72)	9.1974	0.0024	28 (35.90)	20.0998	<0.0001	3 (42.86)	3.1378	0.0765
Cerebrum	83	57.83	0.0059	0.939	68 (10.21)	0.5336	0.4651	5 (5.32)	2.4216	0.1197	9 (11.54)	0.2857	0.593	1 (14.29)	0.1587	0.6903
Cerebellum, NOS	69	94.2	39.9846	<0.0001	38 (5.71)	25.3712	<0.0001	5 (5.32)	1.1428	0.2851	26 (33.33)	72.5845	<0.0001	0 (0)	0.6276	0.4282
Brain stem	42	71.43	3.1678	0.0751	39 (5.86)	5.2195	0.0223	3 (3.19)	0.7086	0.3999	0 (0)	4.4946	0.034	0 (0)	0.3692	0.5434
Ventricle, NOS	11	18.18	7.347	0.0067	10 (1.5)	0.9761	0.3232	0 (0)	1.395	0.2376	1 (1.28)	0.0003	0.9871	0 (0)	0.0931	0.7603
Posterior cranial	1	100	0.7183	0.3967	1 (0.15)	0.2691	0.6039	0 (0)	0.1253	0.7233	0 (0)	0.1018	0.7497	0 (0)	0.0084	0.9271
Fourth ventricle	1	100	0.7183	0.3967	0 (0)	3.7521	0.0536	1 (1.06)	7.9988	0.0047	0 (0)	0.1018	0.7497	0 (0)	0.0084	0.9271
Histology																
Astrocytoma, NOS	394	48.48	28.8372	<0.0001	330 (49.55)	10.7889	0.001	53 (56.38)	4.0449	0.0443	8 (10.26)	45.6775	<0.0001	3 (42.86)	0.0403	0.8409
Astrocytoma, pilocytic	168	90.48	89.6698	<0.0001	96 (14.41)	58.997	<0.0001	18 (19.15)	0.0356	0.8502	51 (65.38)	111.6994	<0.0001	3 (42.86)	2.3391	0.1262
Astrocytoma, anaplastic	154	50	5.2376	0.0221	141 (21.17)	18.3127	<0.0001	8 (8.51)	6.6969	0.0097	5 (6.41)	8.0484	0.0046	0 (0)	1.5731	0.2098
Astrocytoma, fibrillary	56	69.64	3.2146	0.073	52 (7.81)	7.0815	0.0078	1 (1.06)	5.2902	0.0214	3 (3.85)	1.0741	0.3	0 (0)	0.501	0.4791
Astrocytoma, gemistocytic	38	52.63	0.5118	0.4744	36 (5.41)	6.0402	0.014	0 (0)	4.9803	0.0256	2 (2.56)	0.7476	0.3872	0 (0)	0.3324	0.5643
Xanthoastrocytoma, pleomorphic	24	41.67	2.7844	0.0952	0 (0)	91.9065	<0.0001	14 (14.89)	55.6828	<0.0001	9 (11.54)	23.5601	<0.0001	1 (14.29)	3.3507	0.0672
Oligoastrocytoma, anaplastic	4	0	5.6016	0.0179	4 (0.60)	1.0802	0.2987	0 (0)	0.503	0.4782	0 (0)	0.4087	0.5226	0 (0)	0.0336	0.8546
Astrocytoma, protoplasmic	4	0	5.6016	0.0179	4 (0.60)	1.0802	0.2987	0 (0)	0.503	0.4782	0 (0)	0.4087	0.5226	0 (0)	0.0336	0.8546
Astrocytoma, juvenile	2	100	1.4384	0.2304	2 (0.30)	0.5388	0.4629	0 (0)	0.2509	0.6164	0 (0)	0.2039	0.6516	0 (0)	0.0167	0.897
Astrocytoma, diffuse	1	100	0.7183	0.3967	1 (0.15)	0.22691	0.6039	0 (0)	0.1253	0.7233	0 (0)	0.1018	0.7497	0 (0)	0.0084	0.9271

Hispanic patients had higher rates of astrocytoma located in the temporal lobe (31.9% vs. 22.8%, p<0.05) and brain NOS (28.7% vs. 16.1%, p<0.01), and lower rates of brain overlapping (0.0% vs. 6.0%, p=0.01) locations when compared with non-Hispanic patients (Table 2).

Black patients had higher rates of brain NOS (35.9% vs. 15.7%, p<0.01) and cerebellum (33.3% vs. 5.6%, p<0.01) astrocytoma locations, while lower rates of tumor locations in temporal lobe (0.0% vs. 26.2%, p<0.01), parietal lobe (0.0% vs. 5.9%, p=0.02 per Fisher’s exact test), brain overlapping (0.0% vs. 5.9%, p=0.02 per Fisher’s exact test), and brainstem (0.0% vs. 5.5%, p=0.03 per Fisher’s exact test) when compared with astrocytoma locations in non-Black patients (Table 2).

Primary tumor site and vital status

When assessing primary tumor and vitality, frontal lobe had a higher mortality rate compared with other sites (62.4% vs. 36.3%, p<0.01), as well as parietal lobe (71.1% vs. 40.1%, p<0.01) and ventricle (81.8% vs. 41.3%, p=0.01 per Fisher’s exact test). However, a lower mortality rate was observed for brain NOS (31.1% vs. 44.1%, p<0.01) and cerebellum (5.8% vs. 45.0%, p<0.01) when compared with other tumor sites.

Race and tumor histology

White patients had significantly higher rates of astrocytoma NOS (49.6% vs. 35.8%, p<0.01), anaplastic astrocytoma (21.2% vs. 7.3%, p<0.01), fibrillary astrocytoma (7.8% vs. 2.2%, p=0.01), and gemistocytic astrocytoma (5.4% vs. 1.1%, p=0.01), while lower rates of pilocytic astrocytoma (14.4% vs. 40.2%, p<0.01), and pleomorphic xanthoastrocytoma (0.0% vs. 13.4%, p<0.01) when compared with same histology in non-White astrocytoma diagnosed patients, respectively.

Hispanic patients had significantly higher rates of astrocytoma NOS (56.4% vs. 45.4%, p=0.04) and pleomorphic xanthoastrocytoma (14.9% vs. 1.3%, p<0.01 per Fisher’s exact test), while lower rates of anaplastic astrocytoma (8.5% vs. 19.4%, p=0.01), fibrillary astrocytoma (1.1% vs. 7.3%, p=0.02), and gemistocytic astrocytoma (0.0% vs. 5.1%, p=0.02 per Fisher’s exact test), when compared with histology in non-Hispanic population.

Black patients had significantly greater rates of pilocytic astrocytoma (65.4% vs. 15.3%, p<0.01) and pleomorphic xanthoastrocytoma (11.5% vs. 2.0%, p<0.01 per Fisher’s exact test), while lower rates of astrocytoma NOS (10.3% vs. 50.3%, p<0.01) and anaplastic astrocytoma (6.4% vs. 19.4%, p<0.01) compared with non-Black population.

Histology and vital status

Astrocytoma (not otherwise specified) had significantly greater mortality rates compared with all other histology (51.5% vs. 33.3%, p<0.01), along with anaplastic astrocytoma (50.0% vs. 39.9%, p=0.02), anaplastic oligoastrocytoma (100% vs. 41.5%, p=0.03 per Fisher’s exact test), and protoplasmic astrocytoma (100% vs. 41.5%, p=0.03 per Fisher’s exact test), while pilocytic astrocytoma had significantly lower mortality rates when compared with other histology (9.5% vs. 49.8%, p<0.01).

## Discussion

With an average annual age-adjusted incidence rate of 5.42 per 100,000, tumors occurring within the cerebrum constitute the most common neoplasms in children and adolescents from 0-19 years of age [1,18]. Given the nature of CNS neoplasms to confer high degrees of morbidity and mortality, it is increasingly important to continue to elucidate risk factors and prognostic indicators associated with gliomas and other CNS tumors [19,20]. Despite this obvious need, there remains a paucity of reliable data regarding CNS neoplasms, and more thorough quotes of prevalence and survival stratified by grade, age, tumor primary site, and ethnicity are not readily available [18,19]. This study has compiled data that can contribute to the astrocytoma narrative and help shed light on possible trends.

Prevalence

Our prevalence data echoes that of the national epidemiology in that among our sample, White individuals had the highest prevalence (n=666, 78.8%), followed by Hispanics (n=94, 11.1%) and Black individuals (n=78, 9.2%). Among Hispanics nationally, the overall incidence rate for primary brain and CNS tumors is 20.02 per 100,000 and 21.72 per 100,000 among non-Hispanics, with pituitary neoplasms being the only tumor type more frequent in Hispanics than in non-Hispanics. Astrocytoma in particular were calculated to have an incidence of 1.54 per 100,000 in the overall national population between the years 2007 to 2011 [1]. Incidence rates for glioblastoma, astrocytoma (excluding pilocytic), oligodendroglioma, oligoastrocytic tumors, and nerve sheath tumors are approximately two times greater in White individuals than in Black individuals, and pilocytic astrocytoma are also higher among White individuals than Black individuals [1]. Our prevalence data, reflected in Tables 1, 2, seems to follow this national trend of predominantly White patients being affected, with average incidence per 100,000 from 1976 to 2014 of the White population at 1.61 (1.66 nationally), the Hispanic population at 0.56 (1.06 nationally), the Black population at 1.06 (0.86 nationally), and Other at 0.15 [1]. The discrepancy between our Hispanic incidence and that of the national data may be due to differences in parameters for determining Hispanic race, which were mutually exclusive from Black and White in our study. National data did not stratify Hispanics as their own race and as such included Black and White individuals. Statistical analysis of patients in our Central Texas population showed that Hispanic (p=0.00508) and Other race (p=0.01505) patients have significantly lower prevalence rates than White patients. Prevalence rates in our Black patient population showed an increased incidence when compared with Whites, but this difference did not reach statistical significance (p=0.14020).

One possible explanation for these discrepancies in tumor prevalence between racial groups lies within genetic variation. Several studies have found possible differences in genetic pathways to glioma based on race and ethnicity, while other genome-wide studies have found evidence of genomic regions associated with glioma prevalence [17,21]. Since genomes vary from race to race, it can be postulated that the degree of variability in astrocytoma prevalence between ethnic groups may be at least partially attributable to genetic differences that inherently exist between various ethnic groups [22]. One study in particular that focused on the San Francisco Bay Area population found that astrocytomas with p53 mutations and subsequent tumor p53 (TP53) accumulation were more prevalent in non-White than White individuals. Interestingly, however, they documented certain subtypes of astrocytomas that exhibited abnormal TP53 accumulation in the absence of detectable mutations in the p53 gene, thereby indicating that White individuals may be more likely than non-White individuals to develop this particular type of astrocytoma [21].

It could indeed be postulated that various racial groups are unequally exposed to environmental or occupational factors that increase their susceptibility to astrocytoma and other gliomas. To date, studies have failed to find significant correlations between lifestyle exposure and glioma risk with the exception of ionizing radiation, which together with hereditary factors only account for approximately 5% of brain neoplasms [15]. This is one area that researchers have identified as a target for future studies to be conducted. Various occupations may be distributed unequally between races, and thus any occupational exposures linked with the onset of astrocytoma may explain the variance of prevalence among races. Studies examining occupational exposure and prevalence of primary brain malignancies have found conflicting results. One study done in Sweden found that exposure to arsenic and mercury was related to an increased risk of glioma and meningioma in men, and there was a possible association for exposure to petroleum products. However, no such correlations were noted in women [23]. Based on a recent comprehensive review of current epidemiologic data, other studies have inconsistently observed an increased risk of glioma in the following occupations: physicians, firefighters, chemical and other industrial workers, and military personnel [16]. Most of these occupations are primarily taken by people of White ethnicity, and thus is consistent with national prevalence rates of astrocytoma based on race as well as our own. Interestingly, no associations between farming or pesticide exposure and glioma risk were noted in the Upper Midwest Health Study (UMHS) [16]. Future research should aim to elucidate possible environmental factors contributing to astrocytoma prevalence.

Tumor sites

Primary tumor site may also be a significant prognostic factor in astrocytomas. White patients had higher prevalence rates of parietal lobe (6.6% vs. 0.6%, p<0.01), brain overlapping (6.8% vs. 0.0%, p<0.01), and brainstem (5.9% vs. 1.7%, p=0.02) tumors. In our sample, frontal lobe tumors had a higher mortality rate compared to other sites (62.4% vs. 36.3%, p<0.01), as well as parietal lobe (71.1% vs. 40.1%, p<0.01) and ventricle (81.8% vs. 41.3%, p=0.01 per Fisher’s exact test). Given that White individuals suffered a higher mortality rate than Black individuals in our sample, a possible explanation may be due to the disproportionate rate White individuals suffered from tumors located in the parietal lobe while Black patients had higher rates of tumors located in brain NOS (35.9% vs. 15.7%, p<0.01) and cerebellum (33.3% vs. 5.6%, p<0.01), which overall had lower mortality rates compared to other tumor sites (brain NOS 31.1% vs. 44.1%, p<0.01; cerebellum 5.8% vs. 45.0%, p<0.01). Black individuals also experienced lower rates of temporal lobe (0.0% vs. 26.2%, p<0.01) and parietal lobe (0.0% vs. 5.9%, p=0.02 per Fisher’s exact test) tumors, which our study found to have the highest mortality rates. National statistics echo this trend with parietal lobe tumors exhibiting a five-year survival rate of 19.5% and 22.6% for temporal lobe tumors, compared to cerebellar tumors with a survival rate of 71.0% [1]. Hispanic patients had higher rates of tumor located in the temporal lobe (31.9% vs. 22.8%, p<0.05) and brain NOS (28.7% vs. 16.1%, p<0.01) when compared with the remaining sample. These tumor locations failed to achieve significance with regards to mortality. Regardless, it is possible that these tumor locations could achieve statistically significant mortality rates in a larger sample size study. A lower prevalence of neoplasms in more lethal tumor sites and a higher rate of more benign tumor locations could possibly provide an explanation for the relatively lower mortality seen in Black patients with astrocytoma. It is possible that these individuals are more prone to have astrocytoma malignancy in the cerebellum when compared to other populations, and thus present with a much more benign and/or treatable disease process. The genetic pathways described earlier in this study, or other pathways not yet elucidated, could indeed contribute to variance in tumor site location among racial groups and subsequently influence prognostic outcomes of disease.

Tumor histology

Discrepancies in histology between races were also noticed. White patients had significantly higher rates of anaplastic astrocytoma (21.2% vs. 7.3%, p<0.01) when compared with the same histology in non-White astrocytoma diagnosed patients. Hispanic patients had lower rates of anaplastic astrocytoma (8.5% vs. 19.4%, p=0.01), and Black patients had significantly greater rates of pilocytic astrocytoma (65.4% vs. 15.3%, p<0.01) and lower rates of anaplastic astrocytoma (6.4% vs. 19.4%, p<0.01) compared with non-Black population. Anaplastic astrocytoma have a poor five-year relative survival rate at 27.3%, and the prognosis is particularly dismal in patients aged 55 or greater [1,24]. The World Health Organization defines anaplastic astrocytoma as a diffusely infiltrative astrocytic tumor exhibiting characteristic anaplasia and mitotic activity, and as such are regarded as grade III and aggressive [25]. Given this information, it is possible to suspect that the greater incidence of a particularly aggressive tumor in the White population could contribute to more dismal prognosis and mortality rates in that population. Echoing our data, national aggregates also suggests that Black individuals have significantly less incidence of anaplastic astrocytoma (0.40 per 100,000; 95% CI 0.39-0.42 vs. 0.19 per 100,000; 95% CI 0.17-0.21) and a significantly higher incidence of pilocytic astrocytoma (0.36 per 100,000; 95% CI 0.35-0.37 vs. 0.25 per 100,000; 95% CI 0.23-0.27). Pilocytic astrocytoma are known to be quite benign with a five-year survival rate of 94.1%. This could be a source of further exacerbation of the gap between White and Black populations with regards to prognosis and survival.

Mortality and other factors

An interesting finding of this study is the discrepancy in vital status between various ethnic groups. In our study population, Hispanics by far had the largest proportion of deaths (72.3% vs. 38.0%, p<0.01) when compared to the remainder of the sample, followed by White individuals (39.6% vs. 49.7%, p=0.02) and Black individuals (21.8% vs. 43.8%, p<0.01). Our Black patients had the best survival outcome, which is consistent with several studies that have shown that Black individuals have better or equal survival when compared with White individuals [20]. This data, however, is limited in its significance since we could not assess the cause of death, and as such mortality in this study is all-cause included. Thus, we could not calculate accurate -year survival rates. Many other cancers show an opposite trend where Black individuals exhibit a poorer survival rate when compared to White individuals and Hispanics. One study utilizing Surveillance, Epidemiology, and End Results (SEER) Program data found that despite overall improvements in the relative risk of cancer mortality in all racial groups from 1988 to 1997 when compared with 1975 to 1987, the Black sample showed a lower rate of improvement when compared to White individuals (Hispanic and non-Hispanic) and Asian Americans in lung, colorectal, breast, and prostate cancers [26]. Another study utilizing SEER data found that race was not a significant prognostic factor in primary malignant astrocytomas of the spinal cord [27].

Variations in access to healthcare have long been proposed as a significant source of discrepancies among outcomes between different racial groups. Black and Hispanic populations are known to have a higher proportion of individuals living in lower socioeconomic status (SES). Per the US Census Bureau, in 2014, 26% of Blacks and 24% of Hispanics lived below the poverty line. This is in stark contrast to 10% of non-Hispanic Whites living below the poverty line [28]. Individuals of limited socioeconomic standing have exhibited an association with lower overall health care use, regardless of insurance status [28]. Many do not have the financial means to afford insurance and cannot pay out-of-pocket, thus they have limited interaction with the healthcare system, leading to approximately 12% of Blacks and 20% of Hispanics qualifying as uninsured, again in stark contrast to 8% of non-Hispanic Whites [28]. Several studies have shown that standard preventative care is not as frequently accessed by low SES populations, translating into fewer OB/GYN screening procedures, immunizations, diabetic eye examinations, delayed prenatal care, and diminished quality of hospital stays. A substantial amount of evidence and studies evaluated by the Department of Health and Human Services (DHHS) in 1985 and again in 2000 by the CDC and Morehouse School of Medicine, and the Institute of Medicine in 2003 have revealed that many diseases and cancers are not diagnosed at to the same frequency in these populations when compared to the White population [29]. With specific regards to anaplastic astrocytoma, a recent study has shown that individuals with private insurance exhibited greater survival compared to patients on Medicaid, Medicare, or who did not have insurance. Income greater than $63,000 was also shown to be a significant positive prognostic factor, with uninsured status and income less than $38,000 being negative prognostic factors [30]. In Central Texas, these racial discrepancies are also present as they are nationally. Central Texas counties (excluding Travis, Williamson, and Hays counties) have lower median income statistics when compared with other counties in the state, and thus a higher proportion of White individuals in Central Texas are also considered to be of lower socioeconomic status when compared to other regions. This could partially contribute to the more dismal prognosis of astrocytoma in the Central Texas White population [29]. Some studies have also shown that minority groups also receive a lower quality of care even when access to medical services are available [29,30]. Therefore this, coupled with the fact that Texas has a higher proportion of Hispanic individuals when compared with other states, could indeed play a role in the high mortality rate amongst our Hispanic population.

Study limitations

One of the limitations of this study is the sample size. Many variables could not be accurately assessed for significance using statistical methodology due to a limited sample size. This is not surprising given the somewhat limited geographical area of Central Texas. However, the purpose of this study is to add to the collective data pool regarding astrocytoma epidemiology, and thus should be viewed as a meaningful contribution to the current knowledge gap in race and astrocytoma that will benefit from additional sources of information. Another limitation is the collection of data over time. Cases reported in this study range from 1976 to 2014. During this time interval, treatment methods for astrocytomas have evolved, guidelines have changed, and diagnostic techniques have improved. As such, the incidence, prevalence, and cure rates may indeed vary over time. We did not account for such evolution in our analysis and thus there may be confounders to our results. Additionally, access to healthcare has also fundamentally changed, especially since the passing of the Affordable Care Act. As such, minority groups and individuals of lower socioeconomic status that in the past may not have been diagnosed with astrocytoma due to barriers in healthcare access are now being diagnosed, thus affecting the incidence and prevalence of the disease in recent years. Another limitation of the study was the inability to statistically compare our incidence rates to that of national data due to the lack of access to Central Brain Tumor Registry of the United States (CBTRUS) raw data. Future studies will benefit from limiting data to more recent cases and accumulating large sample sizes from multiple regions in order to garner a more updated and accurate picture of astrocytoma epidemiology. Prognostic factors may be more readily elucidated and analyzed as a result. As elucidated earlier, a major limitation of the study that affects prognosis was our inability to elucidate the causes of death. All of the data on mortality in the registry did not include cause of death, and thus it is not known whether the cause of death of expired patients included in the study data was their neoplastic pathology. Future studies would benefit from detailed mortality data in order to calculate five-year survival rates in patients diagnosed with astrocytoma which would allow for a more detailed analysis of prognosis. Treatment modalities were also not addressed in this study. Finally, Black (9.2%) and Hispanic (11.1%) of all tumor patients in this study do not necessarily reflect the general population of Central Texas. This could certainly limit the generalization of the results and conclusion. 

## Conclusions

Several factors could contribute to the pathogenesis and prognosis of astrocytomas. Discrepancies in prevalence of particular histological forms and tumor sites amongst various racial groups may be due to a number of factors. According to current literature, primary tumor sites and tumor histology seem to indeed play a role in mortality and present variably between ethnic groups according to both national statistics and data presented in this study. This may be influenced by discrepancies between races of various factors including genetic predisposition, environmental and occupational exposure, and inequity in access to healthcare resources. Future studies would benefit from collecting a significant amount of more current data, particularly including information on survival and treatment modalities, to undergo stratified analyses in order to better elucidate these possible risk factors and prognostic indicators.
